# Impact of dissolved oxygen and loading rate on NH_3_ oxidation and N_2_ production mechanisms in activated sludge treatment of sewage

**DOI:** 10.1111/1751-7915.13599

**Published:** 2020-06-02

**Authors:** Xueyu Zhang, Shida Li, Shaokui Zheng, Shoupeng Duan

**Affiliations:** ^1^ MOE Key Laboratory of Water and Sediment Sciences/State Key Lab of Water Environment Simulation School of Environment Beijing Normal University Beijing 100875 China

## Abstract

Microaerobic activated sludge (MAS) is a one‐stage process operated at 0.5–1.0 mg l^−1^ dissolved oxygen (DO) aiming at simultaneous nitrification and denitrification. We used molecular techniques and a comprehensive nitrogen (N)‐transformation activity test to investigate the dominant NH_3_‐oxidizing and N_2_‐producing mechanism as well as the dominant ammonia‐oxidizing bacteria (AOB) species in sludge samples individually collected from an MAS system and a conventional anoxic/oxic (A/O) system; both systems were operated at a normal loading rate (i.e. 1.0 kg chemical oxygen demand (COD) m^−3^ day^−1^ and 0.1 kg NH_4_^+^‐N m^−3^ day^−1^) in our previous studies. The DO levels in both systems (aerobic: conventional A/O system; microaerobic: MAS system) did not affect the dominant NH_3_‐oxidizing mechanism or the dominant AOB species. This study further demonstrated the feasibility of a higher loading rate (i.e. 2.30 kg COD m^−3^ day^−1^ and 0.34 kg NH_4_^+^‐N m^−3^ day^−1^) with the MAS process during sewage treatment, which achieved a 40% reduction in aeration energy consumption than that obtained in the conventional A/O system. The increase in loading rates in the MAS system did not affect the dominant NH_3_‐oxidizing mechanism but did impact the dominant AOB species. Besides, N_2_ was predominantly produced by microaerobic denitrification in the MAS system at the two loading rates.

## Graphical abstract

(i) The DO levels in both systems (aerobic: conventional A/O system; microaerobic: MAS system) did not affect the dominant NH3‐oxidizing mechanism or the dominant AOB species. (ii)The MAS process at a high loading rate achieved a 40% reduction in aeration energy consumption than that obtained in the conventional A/O system during sewage treatment. (iii).The increase in loading rates in the MAS system did not affect the dominant NH3‐oxidizing mechanism but did impact the dominant AOB species.

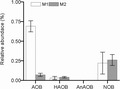

## Introduction

The conventional biological nitrogen removal (BNR) process in municipal wastewater treatment plants (WWTPs), that is the anoxic/oxic (A/O) process, mainly involves aerobic chemolithotrophic ammonia (NH_3_) oxidation by ammonia‐oxidizing bacteria (AOB) or ammonia‐oxidizing archaea (AOA) and nitrite‐oxidizing bacteria (NOB) in the oxic unit; BNR also involves anoxic denitrification by chemoorganoheterotrophic denitrifying microorganisms in the anoxic unit (Dong *et al*., 2017; Zhang *et al*., 2017a). Other microbial N‐transformation pathways, for example aerobic heterotrophic nitrification, anammox and aerobic denitrification, have also been used for N removal in various BNR systems (Guo *et al*., 2014; Pellicer‐Nacher *et al*., 2014; Fitzgerald *et al*., 2015; Borrero‐de Acuna *et al*., 2017; Persson *et al*., 2017; Wen *et al*., 2017; Yang *et al*., 2017; Zhang *et al*., 2017a, 2018; Wang *et al*., 2018). For example, heterotrophic AOB use organic substrates as sources of carbon (C) and energy to convert ammonium (NH_4_^+^) into nitrogen gas (N_2_), and aerobic denitrifying microorganisms conduct denitrification under aerobic conditions (Ren *et al*., 2014; Zhang *et al*., 2017a). These N‐transformation pathways for BNR in wastewater comprise two key steps, namely NH_3_ oxidization, which includes aerobic heterotrophic NH_3_ oxidation, aerobic chemolithotrophic NH_3_ oxidation and anammox; and N_2_ production, which includes anammox, aerobic denitrification and anoxic denitrification (Zhang *et al*., 2017a). Comprehensive N‐transformation activity tests have been conducted to evaluate the contribution of N‐transformation pathways to NH_3_ oxidization and N_2_ production in various BNR processes by determining and comparing the metabolic activities that occur in these pathways (Zhang *et al*., 2017a, 2018). It is now possible to accurately quantify the relative abundance of NH_3_‐oxidizing microorganisms in activated sludge, including AOB (Hallin *et al*., 2005), NOB (Zeng *et al*., 2017), AOA (You *et al*., 2009) and anaerobic AOB (Schmidt *et al*., 2003; Pellicer‐Nacher *et al*., 2014), owing to the development of molecular biological techniques. Furthermore, the community structure and relative abundance of heterotrophic AOB have been estimated using high‐throughput sequencing following isolation and identification techniques for heterotrophic AOB species from activated sludge (Zhang *et al*., 2018). Finally, a combination of relative abundance and metabolic data from various NH_3_‐oxidizing microorganisms are required to elucidate the dominant NH_3_‐oxidizing mechanism for BNR (Zhang *et al*., 2018).

In the conventional A/O process, the oxic and anoxic units are physically separated owing to large differences in their dissolved oxygen (DO) levels. The oxic unit (i.e. the conventional activated sludge process) is characterized by high DO concentrations (> 2 mg l^−1^), high nitrification rates (e.g. > 80%; Liu and Wang, 2013) and low rates of total nitrogen (TN) removal (e.g. < 20%). Conversely, the anoxic unit has low DO levels of < 0.5 mg l^−1^ and transforms nitrification products such as nitrates and nitrites into N_2_, which leads to a TN removal rate of 65–85% during sewage treatment (Ge *et al*., 2014). Our previous investigations demonstrated that simultaneous nitrification and denitrification (SND) can be stably achieved in the conventional activated sludge process solely by modifying its DO concentration to the microaerobic level (0.5–1.0 mg l^−1^), that is the microaerobic activated sludge (MAS) process (Zhang *et al*., 2017b). The MAS process eventually achieves NH_4_^+^‐N and TN removal rates of 99% and 69%, respectively, for sewage treatment when the volumetric loading rates of chemical oxygen demand (COD_LR_; approximately 1.0 kg COD m^−3^ day^−1^) and NH_4_^+^‐N (NH_4_^+^‐N_LR_; approximately 0.1 kg NH_4_‐N m^−3^ day^−1^) in the MAS system (Zhang *et al*., 2017b) are similar to those of the conventional A/O system during sewage treatment (Guo *et al*., 2010; Zhang *et al*., 2017c). However, the potential effects of DO levels (microaerobic vs. aerobic in the MAS and conventional A/O systems) on treatment performance, dominant NH_3_‐oxidizing and N_2_‐producing mechanisms, and dominant AOB species remain unknown. Furthermore, the ability of the MAS system to operate at a higher volumetric loading rate (i.e. higher treatment capacity) and the loading rate effects on the MAS system remain unknown, as we solely operated the system at a normal loading rate in our previous study (Zhang *et al*., 2017b).

Using the conventional A/O system (Zhang *et al*., 2017c, 2018) as a control of the MAS system in our laboratory (Zhang *et al*., 2017b; Phase M1), this study further investigated the potential effect of DO levels (aerobic vs. microaerobic) on treatment performance, overall bacterial communities, dominant NH_3_‐oxidizing and N_2_‐producing mechanisms, and dominant AOB species in both systems at a normal loading rate (i.e. approximately 1.0 kg COD m^−3^ day^−1^ and 0.1 kg NH_4_^+^‐N m^−3^ day^−1^). Notably, these two systems had identical feed characteristics, seed sludge, reactor configurations (biological reaction tank, sedimentation tank, feed pump and reflux pump) and operational parameters (loading rate, reflux ratio, etc.) when the DO levels in the aeration tank were individually maintained at 0.5–1.0 mg l^−1^ for the MAS system and 2.0–3.5 mg l^−1^ for the conventional A/O system (Zhang *et al*., 2017b, 2018). Subsequently, this study demonstrated the feasibility of using a higher volumetric loading rate (i.e. 2.30 kg COD m^−3^ day^−1^ and 0.34 kg NH_4_^+^‐N m^−3^ day^−1^; Phase M2) with the MAS process during sewage treatment. The effects of the two loading rates (normal vs. high) on the treatment performance, overall bacterial communities, dominant NH_3_‐oxidizing and N_2_‐producing mechanisms, and dominant AOB species in the MAS system during sewage treatment were further determined.

## Results and discussion

### Treatment performance of the MAS system at a high loading rate during Phase M2

**Fig. 1:**
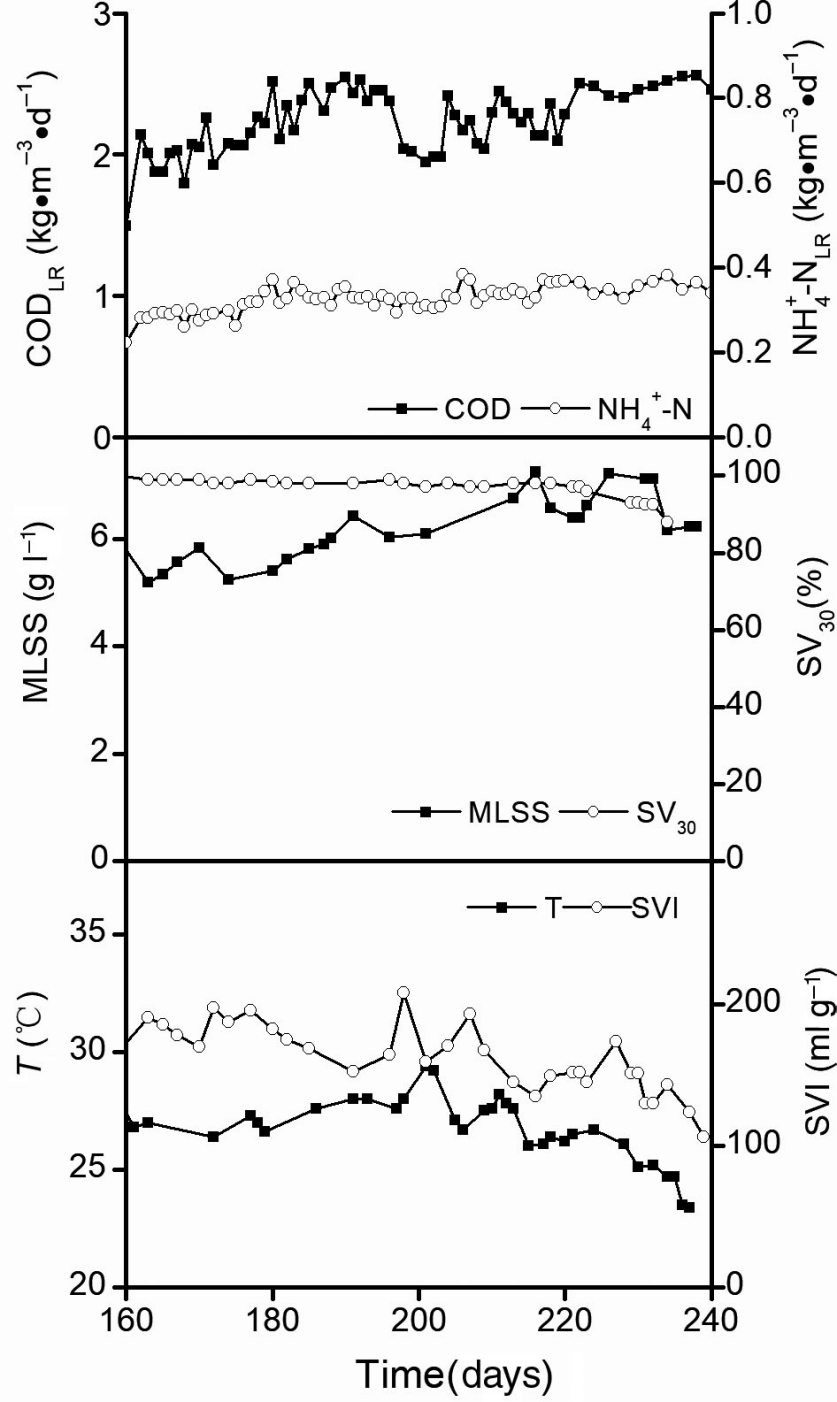
Operational conditions of the laboratory‐scale microaerobic activated sludge (MAS) system at a high loading rate during Phase M2 (days 160–240). Data for Phase M1 (days 1–150) are shown in our previous study (Zhang *et al*., 2017b).

**Fig. 2:**
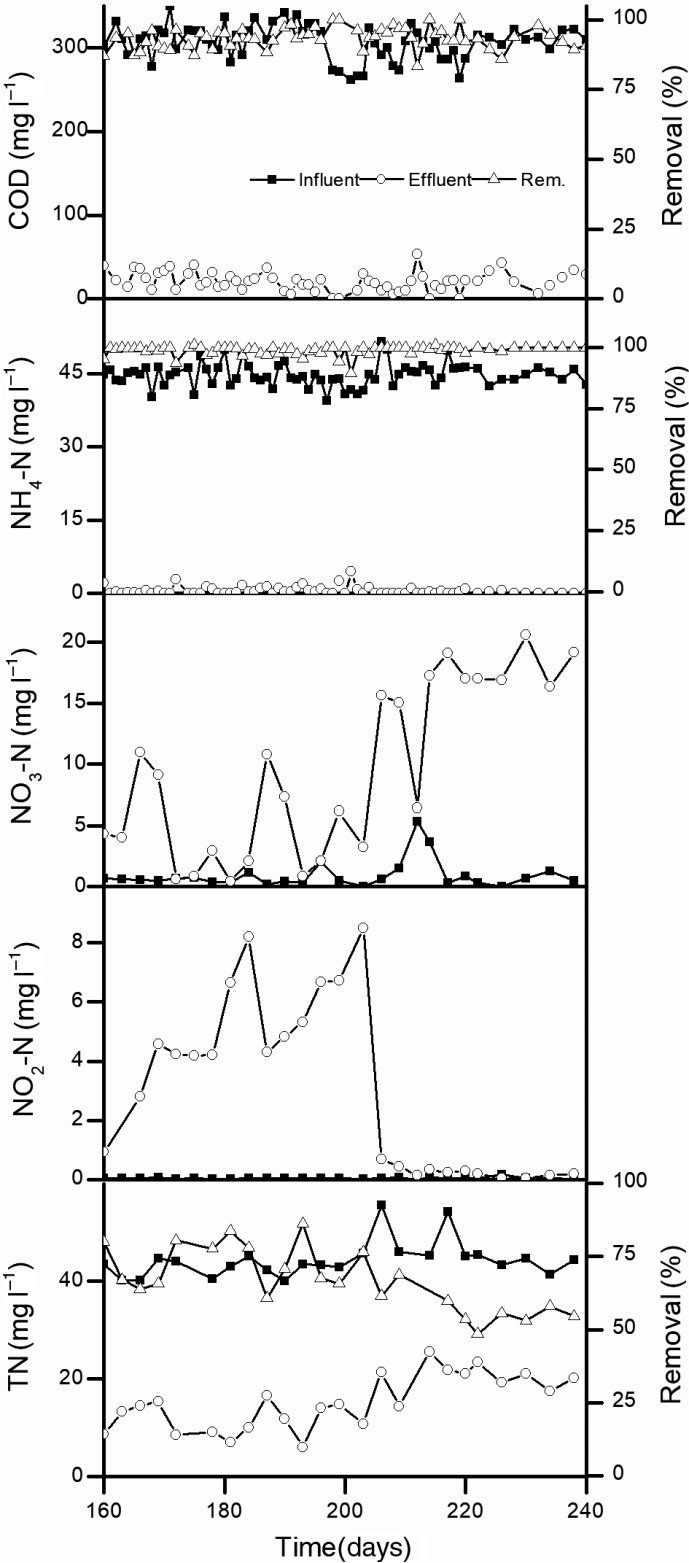
Treatment performance of the laboratory‐scale MAS system at a high loading rate during Phase M2 (days 160–240). Data for Phase M1 (days 1–150) are shown in our previous study (Zhang *et al*., 2017b).

There was a substantial decrease in the hydraulic retention time (HRT) of the aeration tank from 8 h (Zhang *et al*., 2017b; Phase M1: days 1–150) to 3 h (Phase M2: days 160–240) during a total of 240 days of continuous operation of the laboratory‐scale MAS system. The operational conditions and treatment performance of the MAS system during Phase M1 are shown in our previous study (Zhang *et al*., 2017b). In this study, Fig. 1 shows the COD_LR_, NH_4_^+^‐N_LR_, mixed liquor suspended solids (MLSS) concentration, sludge volume index (SVI) and 30 min settling value (SV_30_) results over 80 days of continuous operation of the MAS system during Phase M2. Figure 2 shows the COD, NH_4_^+^‐N, nitrite nitrogen (NO_2_^−^‐N), nitrate nitrogen (NO_3_^−^‐N), and TN levels in the influent and effluent, as well as their removal during Phase M2. The substantial decrease in the HRT of the aeration tank from 8 to 3 h resulted in a significant increase in COD_LR_ and NH_4_^+^‐N_LR_ to 2.30 kg COD m^−3^ day^−1^ and 0.34 kg NH_4_^+^‐N m^−3^ day^−1^, respectively, and an increase in the MLSS level from 2.5–4.4 g l^−1^ (Zhang *et al*., 2017b; Phase M1) to 5.1–7.3 g l^−1^ (Phase M2). The COD_LR_ and NH_4_^+^‐N_LR_ levels of the MAS system during Phase M2 were 2–3 times higher than those of the conventional A/O process (Guo *et al*., 2010; Zhang *et al*., 2017c) for sewage treatment. Throughout the experimental period, the SVI values of the MAS system (130–370 ml g^−1^) approximated those of bulking sludge (150–250 ml g^−1^) found in the conventional A/O system (Guo *et al*., 2013). In view of the narrow range of the SV_30_ levels (92–99%) measured throughout the experimental period, the improvement in SVI values in the MAS system might have been attributed to higher MLSS levels achieved during Phase M2. Under these conditions, COD and NH_4_^+^‐N removal rates averaging 94% and 99%, respectively, were achieved in the MAS system at the two loading rates; these results approximated the removal rates achieved in the conventional A/O control system (COD and NH_4_^+^‐N removal rate averages of 94% and 97% respectively (Zhang *et al*., 2017c)). Additionally, after dividing the amount of COD removed (kg COD removal day^−1^) by the air supply (m^3^ air day^−1^), we calculated the air requirements for COD removal in the conventional A/O control process as 110 ± 19 m^3^ air kg^−1^ of COD removal (Zhang *et al*., 2017c); the air requirements for COD removal in the MAS process during Phase M2 were 66 ± 4 m^3^ air kg^−1^ (data not shown).

No substantial delay in the onset of nitrification was observed in the MAS system during Phase M1 (Zhang *et al*., 2017b) or M2 after the seeded sludge was pre‐cultivated under microaerobic conditions (Fitzgerald *et al*., 2015). High NH_4_^+^‐N‐to‐NO_2_^−^‐N transformation efficiencies are often observed in the BNR process under microaerobic conditions (Liu and Wang, 2013; Zheng *et al*., 2013). However, other researchers have also demonstrated that complete nitrification occurs following long‐term microaerobic operations (e.g. 100 days) after NOB have slowly adapted to the microaerobic environment (Liu and Wang, 2013; Fitzgerald *et al*., 2015; Zhang *et al*., 2017b). In this study, the MAS process during Phase M2 required 30 days to achieve complete nitrification after the maximum NH_4_^+^‐N removal occurred. Similar to other microaerobic BNR processes (Third *et al*., 2003; Zhang *et al*., 2011; Bagchi *et al*., 2012; Zheng *et al*., 2013; Pellicer‐Nacher *et al*., 2014), high SND reaction rates ultimately achieved high TN removal rates via the MAS system during Phase M1 (Zhang *et al*., 2017b) or M2. However, in this study, TN removal (64%) via the MAS system during Phase M2 was lower than that of the MAS system during Phase M1 (72%; Zhang *et al*., 2017b) or the conventional A/O control system (75%; Zhang *et al*., 2017c). In the conventional A/O process, higher reflux ratios (e.g. > 200%) often result in higher TN removal rates owing to the anoxic denitrification reaction (Li *et al*., 2014a). However, in the MAS process, TN removal no longer depends on reflux‐triggered anoxic denitrification owing to the presence of SND. In contrast, a high sludge reflux ratio (i.e. 200% used in this study) might cause side‐effects such as hydraulic shock for sludge settling in the clarifier, thereby leading to inefficient sludge restoration to the aeration tank that might finally lower the TN removal rates, as observed in this study. Therefore, the reflux ratio should be optimized (e.g. 100% or less) to improve TN removal by the MAS system in the future.

These results are the first to demonstrate a significant decrease in the aeration tank HRT (3 h) and increases in the volumetric loading rate (2.30 kg COD m^−3^ day^−1^ and 0.34 kg NH_4_^+^‐N m^−3^ day^−1^) during Phase M2 of the MAS process for sewage treatment compared with the values obtained from the conventional A/O system (i.e. aeration tank HRT: 6–8 h, and volumetric loading rates: 1.00 kg COD m^−3^ day^−1^ and 0.10 kg NH_4_^+^‐N m^−3^ day^−1^). The MAS process during Phase M2 provides numerous advantages over the conventional A/O process, including a 40% reduction in aeration consumption, higher treatment capacity (by 2–3 times) and 60% reduction in aeration tank size requirements. These findings can assist in future designs and operation developments of the MAS process for sewage treatment. However, the need of a larger settling tank owing to sludge bulking will limit wider applications for the MAS process. Therefore, the use of an effective settling process, such as a lamella settler, should be considered and optimized for the MAS process in the future.

### Bacterial communities in the MAS system at the two loading rates

**Fig. 3:**
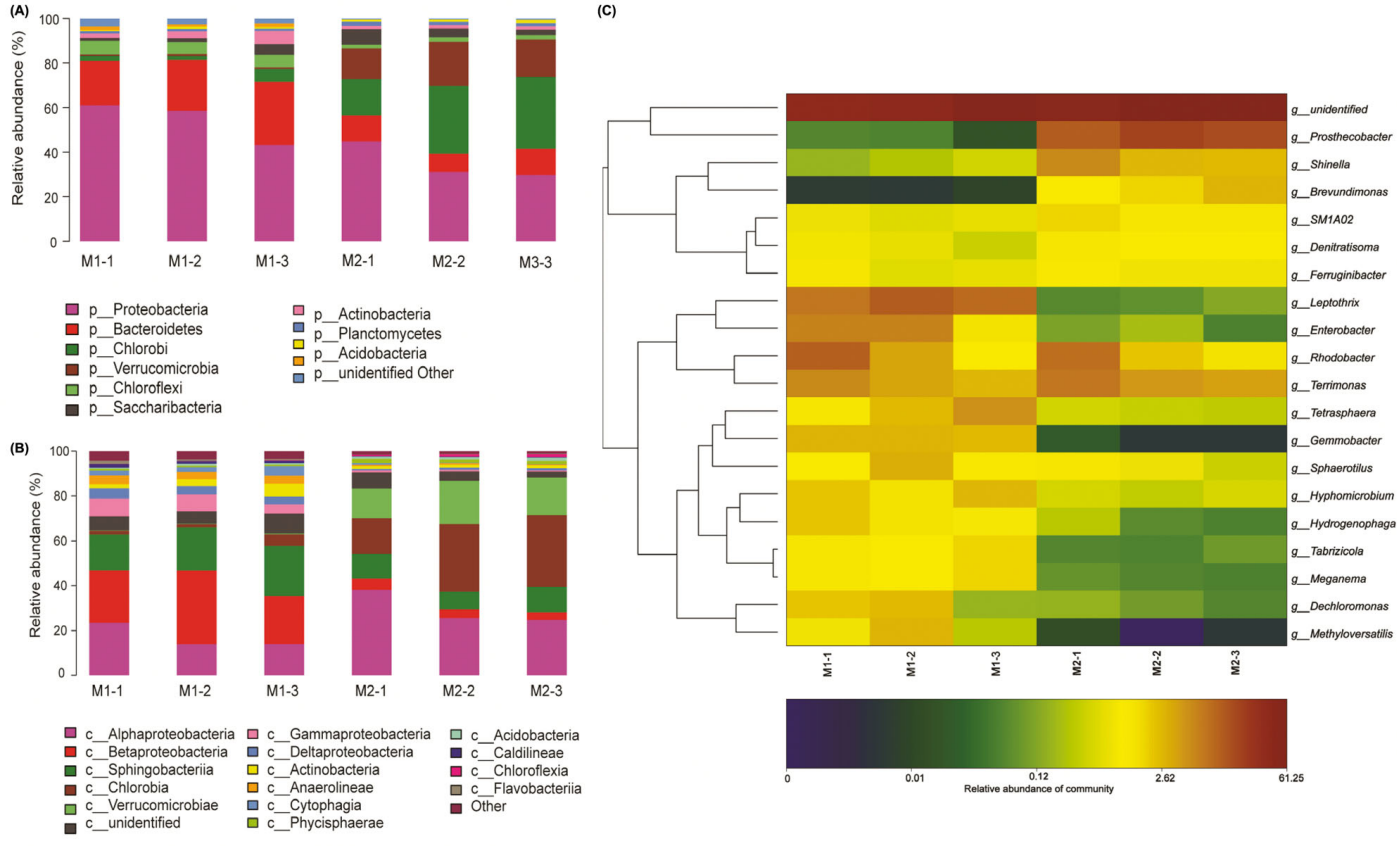
Microbial community structures in six activated sludge samples collected from the MAS system at two loading rates (M1 and M2) at the (A) phylum, (B) class and (C) genus levels based on pyrosequencing. Three sludge samples for each pseudo‐steady‐state period (e.g. M1‐1, M1‐2, and M1‐3 for Phase M1) were collected from the aeration tank every 7 days for high‐throughput pyrosequencing during 14 days of continuous operation.

We analysed the bacterial communities of three sludge samples for each pseudo‐steady‐state period of the MAS system using high‐throughput pyrosequencing. The number of operational taxonomic units (OTUs) observed per sample (30, 604–38, 564 reads) ranged from 236 to 554 (coverage estimate > 0.998). The relative abundances of taxonomic assignments (at the phylum, class and genus levels) in the MAS system at the two loading rates are shown in Fig. 3, while those in the conventional A/O system at a normal loading rate were reported in our previous study (Zhang *et al*., 2018). A previous study found that the microaerobic DO level in an upflow microaerobic sludge blanket reactor promoted the growth and dominance of microaerophilic bacteria (e.g. filamentous bulking bacteria) with a higher O_2_ affinity than that of aerobic bacteria (Zheng *et al*., 2013). Our observations further demonstrated that the difference in DO levels between Phase M1 of the MAS process (microaerobic) vs. the conventional A/O process (aerobic) and the difference in loading rates used in the MAS system led to considerable changes in the overall bacterial community structure at the phylum, class and genus levels in the sludge samples.

**Fig. 4:**
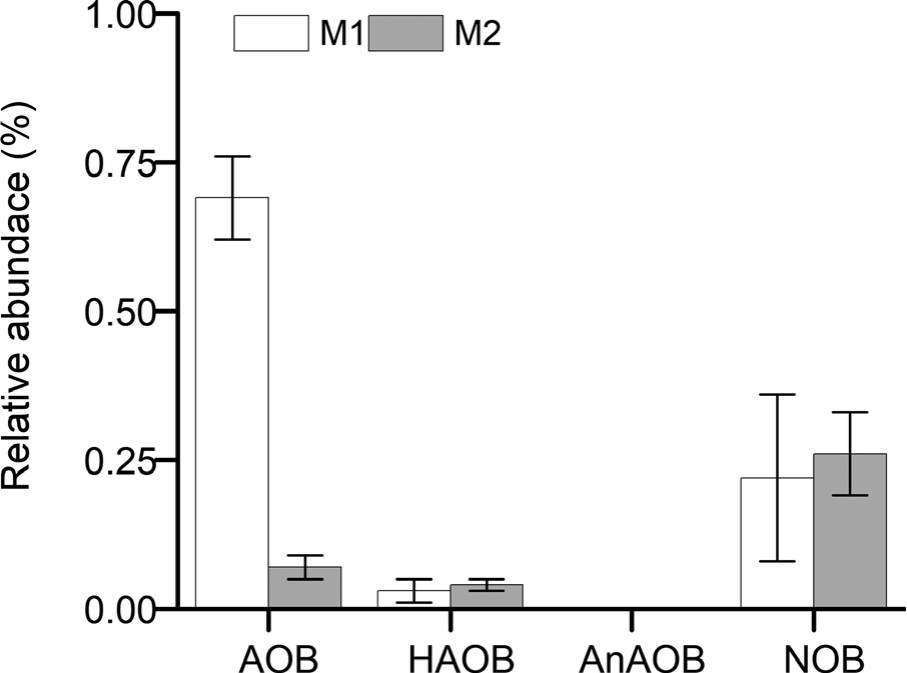
Mean relative abundances of ammonia‐oxidizing bacteria (AOB), nitrite‐oxidizing bacteria (NOB), heterotrophic AOB and anaerobic AOB in sludge samples (three samples for each pseudo‐steady‐state period) collected from the MAS system at two loading rates (M1 and M2) based on pyrosequencing.

Sludge bulking has been described in terms of filamentous bulking and viscous bulking (i.e. non‐filamentous bulking; van den Akker *et al*., 2010). The high‐throughput sequencing results were also used to investigate the relative abundance of bulking and foaming bacteria (BFB) in the conventional A/O system and the MAS system at the two loading rates based on the database for the 16S rRNA gene V3–V4 pyrotags of the BFB groups (Guo and Zhang, 2012). The total percentage of BFB in the conventional A/O control system averaged at only 1.4% (Table S1). The total percentage of BFB present in the bulking sludge was estimated to be 24% in a previous study (Mielczarek *et al*., 2012). In this study, the microaerobic conditions in the MAS system promoted overgrowth of filamentous bacteria with total percentages of BFB as high as 16% during Phase M1; these BFB had dominating proportions of *Leptothrix* (63%), *Tetrasphaera* (19%) and *Sphaerotilus* (12%; Table S1). Light microscopy (Fig. S1) revealed that filamentous bacteria were commonly found in sludge samples collected during Phase M1. However, the changes in the loading rates also appeared to lead to considerable changes in the sludge bulking type (Fig. 4). With the increase in the COD and NH_4_^+^‐N volumetric loading rate, the percentage of BFB in the MAS system during Phase M2 decreased to 2.1% (Table S1). Microscopic observations (Fig. S1) confirmed that filamentous bacteria were less common during this phase. The poor sludge settleability during Phase M2 might have been attributed to viscous bulking caused by insufficient C sources for denitrification (Auterska and Novak, 2006) or excessive production of extracellular polymers (Li *et al*., 2014b).

### Relative abundance of AOB and other ammonia‐oxidizing microorganisms in the MAS system at the two loading rates

#### AOB vs. AOA abundance

Results from the quantitative polymerase chain reaction (qPCR) assays (with three samples per pseudo‐steady‐state period; Table S2) revealed that the relative abundance of AOB NH_3_ monooxygenase subunit A (amoA*)* in the sludge from the MAS system ranged from 7.4 × 10^11^–4.0 × 10^12^ copies g^−1^ of volatile suspended solids (VSS) during Phase M1 to 3.1 × 10^7^–9.2 × 10^7^ copies g VSS^−1^ during Phase M2. By contrast, AOA amoA genes in sludge samples (Table S2) varied within the narrow ranges of < 4.8 × 10^2^ copies μl^−1^ during Phase M1 and 5.0 × 10^6^–2.8 × 10^7^ copies g^−1^ VSS during Phase M2. Thus, the relative abundance of AOB amoA was much higher than that of AOA amoA with abundance ratios of > 2.8 × 10^4^ in all samples. Thus, similar to that in the conventional A/O control system (Zhang *et al*., 2018), aerobic chemolithotrophic NH_3_ oxidation was predominantly conducted by AOB rather than AOA in the MAS system at the two loading rates. AOB are often largely responsible for chemolithotrophic NH_3_ oxidation in various BNR processes because they are more competitive than AOA (Wells *et al*., 2009; Gao *et al*., 2014; Zhang *et al*., 2015).

#### AOB vs. anaerobic AOB abundance

High‐throughput sequencing was used to quantify the AOB, NOB and anaerobic AOB of the MAS system at two loading rates (Table S1) following previously described protocols (Leyva‐Diaz *et al*., 2015a,b; Szabo *et al*., 2017). As observed in the conventional A/O control system (Zhang *et al*., 2018), *Nitrosomonas* also appeared to be the predominant AOB genus in all sludge samples from the MAS system at the two loading rates (Table S1). All detected NOB sequences in the MAS system were classified as *Nitrospira* (57% and 100% during phases M1 and M2 respectively) at the two loading rates (Table S1); however, they were classified as *Candidatus Nitrotoga* (93%) in the conventional A/O control system (Zhang *et al*., 2018). Based on these results (Fig. 4), the mean AOB and NOB abundances in the MAS system at the two loading rates amounted to 0.07–0.69% and 0.22–0.26%, respectively, whereas no anaerobic AOB were detected owing to their low abundance. These values were similar to previously reported results of 0.12–1.00% for AOB and 1.00–2.00% for NOB in biofilm or membrane biological systems, respectively (Leyva‐Diaz *et al*., 2015a,b), and an extremely low anaerobic AOB population that was detected in a microaerobic BNR system (Fitzgerald *et al*., 2015). As observed in the conventional A/O control system (Zhang *et al*., 2018), compared with that of AOB, the contribution of anaerobic AOB to NH_3_ oxidization in the MAS system at the two loading rates appeared to be negligible, which was possibly because of the organic matter in the sewage.

#### AOB and heterotrophic AOB abundance

**Fig. 5:**
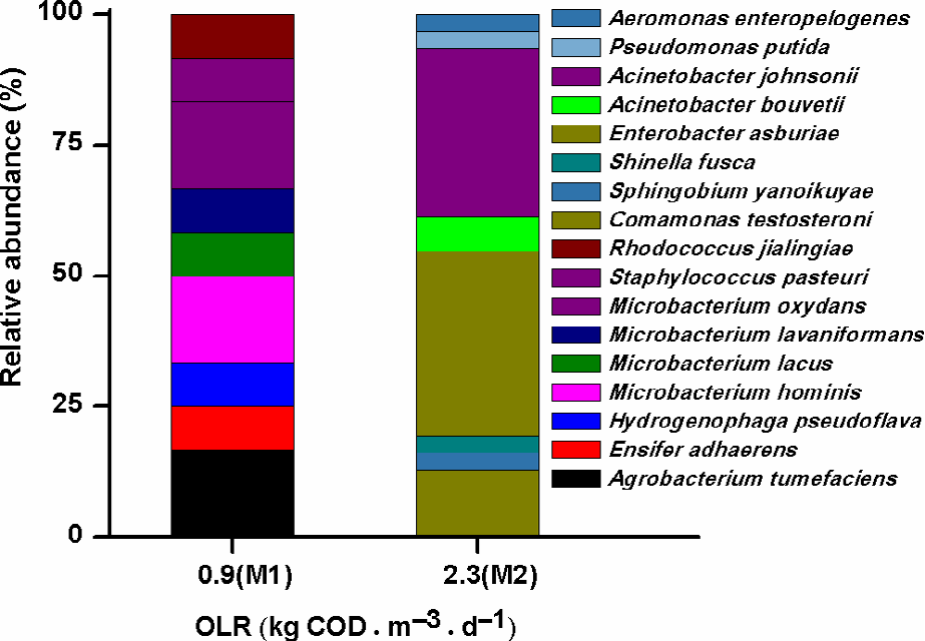
Relative abundance and distribution of operational taxonomic unit (OTU)‐identified isolates used for culturable heterotrophic ammonia‐oxidizing bacteria (AOB) analyses in two libraries from the MAS system at two loading rates (M1 and M2).

We investigated the community structure of culturable heterotrophic AOB in the MAS system at the two loading rates, which included a total of 17 OTUs (Fig. 5). The top three OTUs from the MAS system were identified as *Agrobacterium tumefaciens* (17%), *Microbacterium oxydans* (17%) and *Microbacterium hominis* (17%) during Phase M1, which shifted to *Enterobacter asburiae* (36%), *Acinetobacter johnsonii* (32%) and *Comamonas testosteroni* (13%) during Phase M2. Among them, *A. tumefaciens*, *E. asburiae*, *Pseudomonas putida*, *A. johnsonii* and *Enterobacter cloacae* have been described as capable of both heterotrophic nitrification and aerobic denitrification (Table S3). In this study, the heterotrophic nitrification performances of *A. tumefaciens* and *A. johnsonii*, which are predominant in the two libraries, and *P. putida* were evaluated using an established procedure (Ren *et al*., 2014). The cell growth of these isolates generally reached a plateau within 24 h. These isolates also oxidized NH_4_^+^‐N completely (approximately 90.0 mg l^−1^) and accumulated NO_3_^−^‐N at high levels (1.4–9.2 mg l^−1^; Fig. S2). Thus, these three isolates conducted typical heterotrophic nitrification. Based on the high‐throughput sequencing data, the relative abundances of the genera (e.g. *Pseudomonas* and *Acinetobac*ter) of these heterotrophic AOB isolates were 0.03% and 0.04% in the M1 and M2 sludge samples respectively (Table S1). These values were similar to the proportional abundance of Ammonia‐oxidizing bacteria in these systems (0.07–0.69%). The relative abundance of heterotrophic AOB presented in this study (Fig. 4) might have been overestimated because of other species included in each genus; however, it might also have been underestimated owing to a lack of non‐culturable heterotrophic AOB species in the sludge samples. These results demonstrate that both AOB and heterotrophic AOB contribute to NH_3_ removal in the MAS system at the two loading rates, as observed in the conventional A/O control system (Zhang *et al*., 2018).

### Dominant NH_3_‐oxidizing and N_2_‐producing mechanisms in the MAS system at the two loading rates

**Fig. 6:**
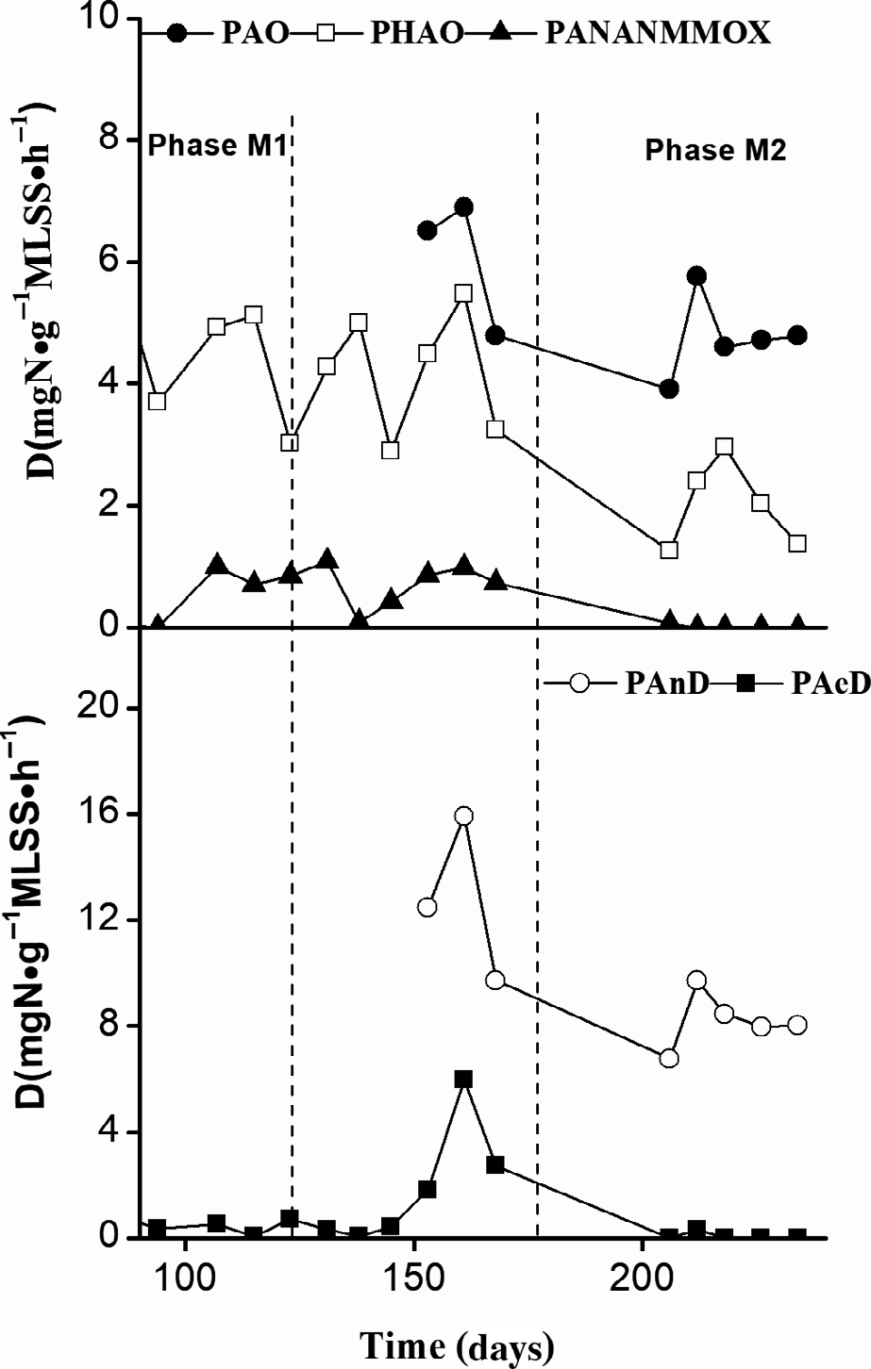
N‐transformation activity characteristics of activated sludge samples collected from the MAS system. D: potential N‐transformation activities; PAO: potential aerobic chemolithotrophic ammonia oxidation activity; PHAO: potential aerobic heterotrophic ammonia oxidation activity; PAnammox: potential anammox activity; PAnD: potential anoxic denitrification activity; PAeD: potential aerobic denitrification activity. The PAO and PAnD activity rates during Phase M1 are shown in our previous study (Zhang *et al*., 2017b).

The changes in various N‐transformation activity levels in the sludge samples during continuous operation of the MAS system at the two loading rates are illustrated in Fig. 6. The potential chemolithotrophic ammonia oxidation (PAO) and anoxic denitrification (PAnD) activity rates during Phase M1 are shown in our previous study (Zhang *et al*., 2017b). The PAO and PAnD activity rates ranged from 3.3 mg N g MLSS^−1^ h^−1^ to 5.2 mg N g MLSS^−1^ h^−1^ and from 6.8 mg N g MLSS^−1^ h^−1^ to 14.1 mg N g MLSS^−1^ h^−1^, respectively, across all sludge samples, and were similar to previously reported values (Tanaka *et al*., 2003; Hallin *et al*., 2005; Morgan‐Sagastume *et al*., 2008).

#### Dominant NH_3_‐oxidizing mechanism

The potential anammox (PAnammox) activity rates in all sludge samples were nearly negligible (< 1.0 mg N g MLSS^−1^ h^−1^), whereas the potential aerobic heterotrophic ammonia oxidation (PHAO) activity rates were relatively high (1.3–5.1 mg N g MLSS^−1^ h^−1^). The metabolic activity rates of AOB, heterotrophic AOB and anaerobic AOB in the MAS system at the two loading rates corresponded to their relative abundance. As observed in the conventional A/O control system (Zhang *et al*., 2018), NH_3_ oxidization was predominantly achieved by a combination of aerobic heterotrophic and chemolithotrophic NH_3_ oxidization in the MAS system at the two loading rates. Therefore, neither the loading rates used in the MAS system nor the change from aerobic (conventional A/O process) to microaerobic (Phase M1 of the MAS process) DO levels affected the dominant NH_3_‐oxidizing mechanism in the sludge samples. Additionally, the PHAO activity rates during Phase M2 of the MAS process (64% of PAO activity) were significantly lower than the activity rates during Phase M1 (92–100% of PAO activity).

#### Dominant N_2_‐producing mechanism

Similar to the results from studies of municipal WWTPs (Wang *et al*., 2015) or the conventional A/O control system (Zhang *et al*., 2018), anammox also played a minor role in N_2_ production in both systems in this study (0.0–7.1% of PAnD activity). Similarly, the potential aerobic denitrification (PAeD) activity rates in the MAS system at the two loading rates were also low (0.0–7.6% of PAnD activity). Anoxic denitrification might have produced most of the N_2_ in the conventional A/O control system, as a 200% reflux ratio and anoxic unit were observed (Zhang *et al*., 2018). However, the MAS system did not have an anoxic zone. Some aerobic denitrifying bacteria exhibit greater activity rates under anoxic rather than oxic conditions (Zhang *et al*., 2017a). Denitrifying enzymes have different activity levels under oxic and anoxic conditions, and the production of denitrifying enzymes shows a negative correlation with the O_2_ pool (Patureau *et al*., 2000). Thus, microaerobic DO levels such as those in the MAS system may also trigger denitrification reactions. In other words, N_2_ in the MAS system was predominantly produced through microaerobic denitrification.

### AOB communities in the MAS system at the two loading rates

**Fig. 7:**
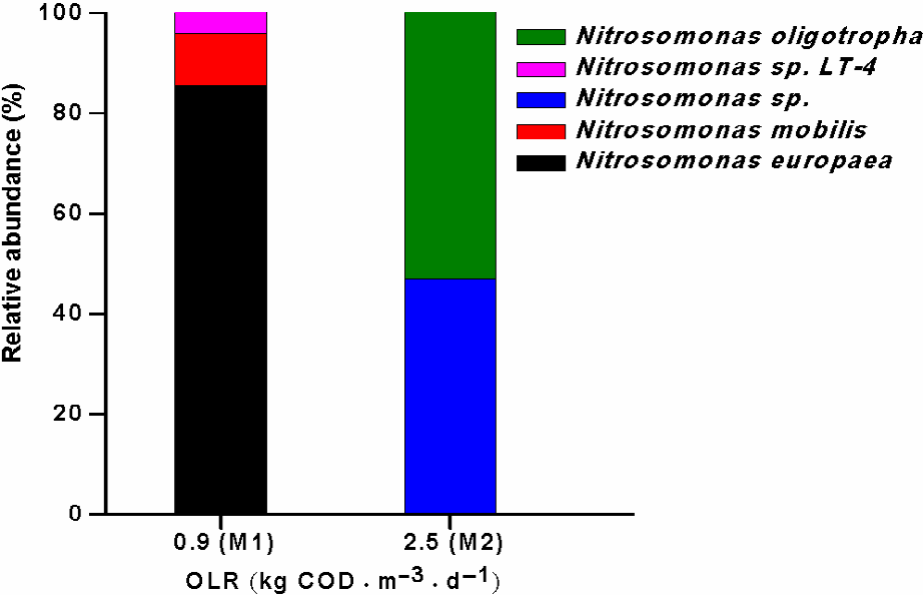
Species composition and relative abundances of AOB clone libraries of the MAS system at two loading rates (M1 and M2). The ammonia monooxygenase subunit A (amoA) gene fragment of AOB was polymerase chain reaction (PCR)‐amplified from genomic DNA using the primer set amoA‐1F/amoA‐2R.

Two AOB clone libraries were constructed for each phase (M1 and M2) of the MAS system. Samples were collected over two pseudo‐steady‐state periods. A total of 97 AOB sequences were classified into 8 OTUs. Coverage estimates of 94–100% indicated that the sequenced clones represented most of the present AOB species. The AOB species distributions and relative abundances in the MAS system at the two loading rates are shown in Fig. 7.

In a previous investigation of eight WWTPs, *Nitrosomonas europaea* was the dominant AOB species (55–67%) in some sludge samples, whereas *Nitrosomonas oligotropha* was predominant (48–97%) in other sludge samples (Gao *et al*., 2013). Previous researchers have often attributed the predominance of these two AOB species to the differences in NH_3_ or DO levels in various BNR systems; this is because these two AOB species have different NH_3_ (Koops and Pommerening‐Roser, 2001; Park and Noguera, 2007) or O_2_ affinities (Park and Noguera, 2004, 2007). In this study, similar to the results in the conventional A/O control system (Zhang *et al*., 2018), the AOB communities in the sludge sampled from Phase M1 of the MAS system were dominated by *N. europaea/eutropha* (86%) and *Nitrosomonas mobilis* (10%; Fig. 7). However, the AOB community in the MAS system during Phase M2 was dominated by *N. oligotropha* (53%) and *Nitrosomonas* sp. (47%), which is an OTU classified within the broadly defined *N. oligotropha* lineage (Fig. 7). The change in the DO levels (microaerobic vs. aerobic in the MAS system during Phase M1 and the conventional A/O control system) might not have led to significant changes in the dominant AOB species in the two sludge samples. Our results further indicated that the significant change in dominant AOB species was observed at the two loading rates (Phases M1 and M2) owing to the significant change in HRT when no significant changes in NH_3_ and DO levels occurred in the activated sludge. Therefore, this study demonstrates that the changes in either NH_3_ or DO levels in various BNR systems may not explain the predominance of these two AOB species in activated sludge. Future studies should be conducted to investigate other niche differentiations (e.g. HRT) of these two AOB species in activated sludge based on the differences in their physiological characteristics (e.g. growth rate). A previous study reported that the maximum specific growth rate of AOB varies in a wide range of 0.02–0.09 h^−1^ (i.e. a generation time of 8–36 h; Vadivelu *et al*., 2006). Under these conditions, the change in dominant AOB species in the MAS system during the two phases might have been attributed to the differences in their growth rates because the change occurred at a significantly shorter HRT. Additionally, Wells *et al*. (2009) reported that the *Nitrosomonas*‐like phylotype showed no significant correlation with the DO levels, while the *Nitrospira* lineage showed strong negative correlations with the DO levels. This result explains why the change in the DO levels (aerobic vs. microaerobic) in both systems did not significantly change the dominance of the *Nitrosomonas*‐like phylotype in the AOB community structure.

### Implications for applications of the MAS process at a high loading rate

As demonstrated in this study, the variation in DO levels led to significant changes in the overall bacterial community structure in the sludge samples. Therefore, the sludge cultivation of the MAS process at a high loading rate should be conducted under microaerobic conditions throughout all the operational phases to achieve successful operation. Furthermore, the increase in the loading rates in the MAS system affected the overall bacterial community structure or the dominant AOB species in the sludge samples and led to a substantial delay in the onset of complete nitrification. Under these conditions, a long operational time will be required to achieve complete nitrification using the MAS process at a high loading rate. To accelerate sludge cultivation, the dominant AOB species and NOB species should be isolated in the future to collect the required amount of dominant AOB and NOB under aseptic conditions in order to accelerate the MAS process start‐up at a high loading rate.

## Conclusions

Phase M2 of the MAS process was characterized by a 3 h aeration tank HRT, COD_LR_ of 2.30 kg COD m^−3^ day^−1^, and NH_4_^+^‐N_LR_ of 0.34 kg NH_4_^+^‐N m^−3^ day^−1^. These characteristics offer several advantages over the conventional A/O control system, including a lower aeration consumption, higher treatment capacity and smaller size requirements for the biological reaction unit. Changes in the DO and loading rates led to significant changes in the overall bacterial community structure in the activated sludge. During the MAS process, SND was achieved by simultaneous aerobic heterotrophic and chemolithotrophic NH_3_ oxidization in combination with microaerobic denitrification. Neither the loading rates used in the MAS system nor the change from aerobic (conventional A/O process) to microaerobic (Phase M1 of the MAS process) DO levels affected the dominant NH_3_‐oxidizing mechanism in the sludge samples. Changes in the loading rates (or HRTs) also led to significant changes in the AOB community structure of the MAS system. Our findings can contribute to the future design and operational development of a high loading level MAS process for sewage treatment.

## Experimental procedures

As described in our previous study (Zhang *et al*., 2017b), the MAS system comprised a 5.8 l lucite aeration tank and a sedimentation tank (Fig. S3). Synthetic wastewater (200–300 mg l^−1^ COD, 45 mg l^−1^ NH_4_^+^‐N and 7 mg l^−1^ total phosphorus) was prepared by dissolving glucose, monopotassium phosphate, ammonium chloride and sodium bicarbonate in tap water without adding any micronutrients. Air was introduced to the aeration tanks via bubble diffusers to maintain the DO level at 0.5–1.0 mg l^−1^ for the MAS system by manually adjusting the airflow rate 3–4 times daily.

After 150 days of continuous operation of the MAS system during Phase M1 (i.e. at COD_LR_ and NH_4_^+^‐N_LR_ levels of 1.00 kg m^−3^ day^−1^ and 0.14 kg m^−3^ day^−1^ respectively) in our previous study (Zhang *et al*., 2017b), the COD_LR_ and NH_4_^+^‐N_LR_ of the MAS system in this study further increased to 2.30 kg m^−3^ day^−1^ and 0.34 kg m^−3^ day^−1^, respectively, for the next 80 days of continuous operation (22–28 °C) during Phase M2 (i.e. days 160–240). We defined the pseudo‐steady‐state period as the time interval in which the effluent pollutant levels during Phase M1 or Phase M2 changed < 10% over three consecutive samples. After the pseudo‐steady‐state period began, activated sludge was collected from the aeration tank every 7 days to immediately evaluate the comprehensive N‐transformation activities, including PAO, PAnammox, PHAO, PAeD and PAnD. When the N‐transformation activities were stable (approximately 10 days after the pseudo‐steady‐state period began), three biomass samples (named M1‐1, M1‐2 and M1‐3 or M2‐1, M2‐2 and M2‐3) were collected from the aeration tank every 7 days for qPCR analysis and high‐throughput pyrosequencing during 14 days of continuous operation. Finally, at the end of the pseudo‐steady‐state period, heterotrophic AOB were isolated from a fresh sludge sample and identified and evaluated using an established procedure (Ren *et al*., 2014; Zhang *et al*., 2018), while the AOB diversity in the fresh sludge sample was determined by constructing a clone library. The identified heterotrophic AOB species were used to evaluate the community structure and relative abundance of heterotrophic AOB in the activated sludge based on the high‐throughput sequencing results (Zhang *et al*., 2018).

The experimental procedures for the N‐transformation activity tests and molecular analyses followed those described previously (Zhang *et al*., 2017a, 2018). The bacterial 16S rRNA gene sequences produced by high‐throughput pyrosequencing were deposited in the NCBI database (accession number SRP130704), and sequences of the AOB amoA genes and heterotrophic AOB 16S rRNA genes were submitted to the GenBank database (accession numbers MG831208–MG831304 and MH064211–MH064253 respectively).

## Conflict of interest

The authors declare that there is no conflict of interests.

## Ethical approval

This article does not contain any studies with animals performed by any of the authors.

## Supporting information


**Table S1.** The relative abundance of ammonia‐oxidizing bacteria (AOB), nitrite‐oxidizing bacteria (NOB), heterotrophic AOB, anaerobic AOB and filamentous bacteria (FB) in different sludge samples collected during two experimental phases (M1 and M2) of the microaerobic activated sludge (MAS) process at normal (M1) and high (M2) loading rates, and during one experimental phase of the conventional anoxic/oxic (A/O) process at a normal loading rate, based on high‐throughput sequencing data.
**Table S2.** Calculated numbers of AOB and ammonia‐oxidizing archaea (AOA) in the sludge samples of the MAS system during two experimental phases (M1 and M2), based on the amoA gene and Arch amoA gene.
**Table S3.** Heterotrophic nitrification performance of all OTU‐identified isolates from sludge samples collected during the two experimental phases (M1 and M2) of the MAS system.
**Fig. S1.** The microscopic appearance of sludge samples collected from the MAS process in Phase M1(a) and Phase M2(b).
**Fig. S2.** Temporal variation of nitrogen compounds concentration and cell growth of OTU‐identified isolates. OD: optical density.
**Fig. S3.** The microaerobic activated sludge system used in this study.
