# Supplementary material for: Impact of dissolved oxygen and loading rate on NH3 oxidation and N2 production mechanisms in activated sludge treatment of sewage
Source: Microb Biotechnol. 2020 Jun 2;14(2):419–29. doi: 10.1111/1751-7915.13599 (PMC7936313; doi:10.1111/1751-7915.13599)
Supplement: Supplementary file 1 — Table S1. The relative abundance of ammonia‐oxidizing bacteria (AOB), nitrite‐oxidizing bacteria (NOB), heterotrophic AOB, anaerobic AOB and filamentous bacteria (FB) in different sludge samples collected during two experimental phases (M1 and M2) of the microaerobic activated sludge (MAS) process at normal (M1) and high (M2) loading rates, and during one experimental phase of the conventional anoxic/oxic (A/O) process at a normal loading rate, based on high‐throughput sequencing data. Table S2. Calculated numbers of AOB and ammonia‐oxidizing archaea (AOA) in the sludge samples of the MAS system during two experimental phases (M1 and M2), based on the amoA gene and Arch amoA gene. Table S3. Heterotrophic nitrification performance of all OTU‐identified isolates from sludge samples collected during the two experimental phases (M1 and M2) of the MAS system. Fig. S1. The microscopic appearance of sludge samples collected from the MAS process in Phase M1(a) and Phase M2(b). Fig. S2. Temporal variation of nitrogen compounds concentration and cell growth of OTU‐identified isolates. OD: optical density. Fig. S3. The microaerobic activated sludge system used in this study. [file MBT2-14-419-s001.docx]

Supplementary Information for

Impact of dissolved oxygen and loading rate on NH_3_ oxidation and N_2_ production mechanisms in activated sludge treatment of sewage

Xueyu Zhang, Shida Li, Shaokui Zheng^[[1]](#footnote-1)^*, Shoupeng Duan

*MOE Key Laboratory of Water and Sediment Sciences/State Key Lab of Water Environment Simulation, School of Environment, Beijing Normal University, Beijing 100875, China*

* Corresponding author.

*E-mail* address: [zsk@bnu.edu.cn](mailto:zsk@bnu.edu.cn) (S. Zheng).

**Summary:**

13 pages, including 3 Tables, 3 Figures, and isolation and identification of heterotrophic AOB in sludge.

**Isolation and identification of heterotrophic AOB in sludge**

**Media** The heterotrophic AOB screening medium (HSM) consisted of the following components (g·L^-1^): (NH_4_)_2_SO_4_ (0.47) and sodium succinate (5.49), together with 50 mL trace elements solution; the pH was adjusted to 7.5. The trace elements solution contained (g·L^-1^): K_2_HPO_4_ (5), MgSO_4_·7H_2_O (2.5), NaCl, (2.5), MnSO_4_·4H_2_O (0.05), and FeSO_4_·7H_2_O (0.05). Solid media were prepared by adding 2% agar to these liquid media.

**Isolation and identification of pure cultures from environmental samples**. Activated sludge samples were taken from the aerobic tank of activated sludge reactors. Following a serial dilution of 10^-1^–10^-9^ with aseptic physiological saline (0.85% NaCl), 0.1 mL of each dilution was transferred in duplicate onto HSM agar plates and cultivated at 30°C for ~3 days under aseptic conditions. Then 50 colonies (>80% of the total colonies on an agar plate) were randomly selected for each sample, and were further purified by repeated streak plate isolation on fresh HSM agar plates. Total genomic DNA from each pure culture was extracted and purified using the OMG-Soil-DNA-Kit (Omega Bio-tek, Inc., USA). The 16S rRNA gene was amplified by PCR from genomic DNA using the universal primers 27f (5′-AGAGTTTGATCMTGGCTCAG-3′) and 1492r (5′-TACGGYTACCTTGTTACGACTT-3′) (19) and sequenced by Majorbio Bio-pharm Technology Co., Ltd. (Beijing, China). All 16S rDNA sequences thus obtained were checked for chimeric artifacts using the Chimera Check program (version 2.7) from the Ribosomal Database Project.

The resulting nucleotide sequences (1260–1510 bp) were aligned using MEGA 6.0 software and then divided into different operational taxonomic units (OTUs) with a 2% distance criterion using DOTUR software. The sequences of these OTUs were compared to those in the database of the National Center for Biotechnology Information to identify closely related bacterial sequences.

**Assessment of heterotrophic nitrification of all OTU-identified isolates** Following activation in the HSM medium for 24 h, 0.5 mL cell suspension from each potential heterotrophic AOB species was individually inoculated into 100 mL sterile HSM medium in a 250 mL Erlenmeyer flask, and was incubated at 30°C and 150 rpm under aseptic conditions, along with uninoculated flasks used as controls. Samples were taken periodically to determine cell growth using spectrophotometry at 600 nm (OD_600_), and the concentrations of NH_4_^+^**-**N, NO_2_**^-^-**N, and NO_3_**^-^-**N were determined according to standard methods (20). For isolates that grew extremely slowly in the HSM medium (e.g., no visual growth after 3 days), the inoculum was harvested by cultivation in 100 mL LB medium, centrifugation at 7000 rpm for 5 min and washing with aseptic physiological saline three times to produce enough biomass for assessment tests. All tests were conducted in duplicates or even triplicates.

**Table S1.** The relative abundance of ammonia-oxidizing bacteria (AOB), nitrite-oxidizing bacteria (NOB), heterotrophic AOB, anaerobic AOB, and filamentous bacteria (FB) in different sludge samples collected during two experimental phases (M1 and M2) of the microaerobic activated sludge (MAS) process at normal (M1) and high (M2) loading levels, and during one experimental phase of the conventional anoxic/oxic (A/O) process at a normal loading level, based on high-throughput sequencing data.

| Communities | OTUs | Closest match | | | Relative abundance(average) | | |
| --- | --- | --- | --- | --- | --- | --- | --- |
|  |  |  |  |  | A/O | M1 | M2 |
| AOB | OTU_97, OTU_580, OTU_207, OTU_372, OTU_194, OTU_444, OTU_552, OTU_704 | g__Nitrosomonas; s__unidentified | | |  | 0.69% | 0.07% |
| Anaerobic AOB | / |  | | |  | ND | ND |
| Heterotrophic AOB | OTU_286; OTU_671 | g__Pseudomonas; | | |  | 0.0% | 0.0% |
|  | OTU_320, OTU_489 | g__Acinetobacter | | |  | 0.03% | 0.04% |
| NOB | OTU_23 OTU_832  OTU_996 | | g__Nitrospira; | |  | 0.13%% | 0.26% |
|  | OTU_70  OTU_886 | | g__Candidatus_Nitrotoga; | |  | 0.10% | 0.2% |
| FB | OTU_320; OTU_489 | | g__Acinetobacter | | 0.08% | 0.03% | 0.04% |
|  | OTU_113 | | g__Bellilinea | | 0.07% | 0.07% | 0.00% |
|  | OTU_273, OTU_579, OTU_209, OTU_486 | | g__Candidatus_Microthrix | | 0.02% | 0.01% | 0.11% |
|  | OTU_677 | | g__Chryseobacterium | | 0.01% | 0.00% | 0.00% |
|  | OTU_196, OTU_720, OTU_129 | | g__Flavobacterium | | 0.07% | 0.00% | 0.16% |
|  | OTU_388, OTU_380 | | | g__Haliscomenobacter | 0.05% | 0.04% | 0.00% |
|  | OTU_6 | | | g__Leptothrix | 0.25% | 10.27% | 0.09% |
|  | OTU_621 | | | g__Mycobacterium | 0.00% | 0.01% | 0.00% |
|  | OTU_82 | | | g__Runella | 0.03% | 0.16% | 0.27% |
|  | OTU_45 | | | g__Sphaerotilus | 0.64% | 1.87% | 0.86% |
|  | OTU_15 | | | g__Tetrasphaera | 0.03% | 3.13% | 0.38% |
|  | OTU_566, OTU_70 | | | g__Thiothrix | 0.01% | 0.69% | 0.19% |
|  | OTU_145 | | | g__Trichococcus | 0.14% | 0.01% | 0.00% |

Note: ND: Not detected; OTU: operational taxonomic unit..

**Table S2.** Calculated numbers of AOB and ammonia-oxidizing archaea (AOA) in the sludge samples of the MAS system during two experimental phases (M1 and M2), based on the amoA gene and Arch amoA gene.

| Samples | OLR  (kg COD·m^-3^ ·d^-1^) | amoA gene  (copies·g^−1^ VSS) | Arch amoA gene  (copies·g^−1^ VSS) | AOB/AOA ratios |
| --- | --- | --- | --- | --- |
| M1_1 | 0.90 | 4.04×10^12^±2.04×10^11^ | 6.06×10^6^±4.20×10^6^ | 8.99×10^5^ |
| M1_2 | 0.90 | 3.14×10^12^±1.37×10^11^ | 5.03×10^6^±1.86×10^6^ | 6.82×10^5^ |
| M1_3 | 0.90 | 7.44×10^11^±5.68×10^10^ | 2.81×10^7^±3.25×10^6^ | 2.77×10^4^ |
| M2_1 | 2.3 | 3.06×10^7^±5.26×10^6^ | ND | / |
| M2_2 | 2.3 | 9.18×10^7^±6.89×10^7^ | ND | / |
| M2_3 | 2.3 | 3.56×10^7^±5.26×10^6^ | ND | / |

Note: OLR: Organic loading rate; ND: Not detected.

**Table S3.** Heterotrophic nitrification performance of all OTU-identified isolates from sludge samples collected during the two experimental phases (M1 and M2) of the MAS system.

| Libraries | OTU | Closest Relative sequence | Relative abundance (%) |  | Relative information | | |
| --- | --- | --- | --- | --- | --- | --- | --- |
|  |  |  |  |  | HN | AD | Reference |
| M1 | 1 | *Agrobacterium tumefaciens* | 16.7 |  | + | + | [4] |
|  | 2 | *Ensifer adhaerens* | 8.3 |  | / | / |  |
|  | 3 | *Hydrogenophaga pseudoflava* | 8.3 |  | / | / |  |
|  | 4 | *Microbacterium hominis* | 16.7 |  | / | / |  |
|  | 5 | *Microbacterium lacus* | 8.3 |  | / | / |  |
|  | 6 | *Microbacterium lavaniformans* | 8.3 |  | / | / |  |
|  | 7 | *Microbacterium oxydans* | 16.7 |  | / | + | [5] |
|  | 8 | *Staphylococcus pasteuri* | 8.3 |  | / | / |  |
|  | 9 | *Rhodococcus jialingiae* | 8.3 |  | / | / |  |
| M2  (31 sequences;  Coverage:87%;  Chao 1 value: 11;  Shannon index: 1.62;  Simpson 0.23) | 1 | *Sphingobium yanoikuyae* | 3.2 |  | / | / |  |
|  | 2 | *Shinella fusca strain DC-196* | 3.2 |  | / | / |  |
|  | 3 | *Comamonas testosteroni* | 12.9 |  | / | / |  |
|  | 4 | *Enterobacter asburiae* | 35.5 |  | + | + | [6] |
|  | 5 | *Acinetobacter johnsonii* | 32.3 |  | + | + | [2] |
|  | 6 | *Acinetobacter bouvetii* | 6.5 |  | / | / |  |
|  | 7 | *Pseudomonas putida* | 3.2 |  | + | + | [7] |
|  | 8 | *Aeromonas enteropelogenes* | 3.2 |  | / | / |  |

^*^ The seed for the assessment was harvested by the cultivation in LB medium. HN: heterotrophic nitrification; AD: aerobic denitrification; +/-: Positive/Negative; /: No data available.

| 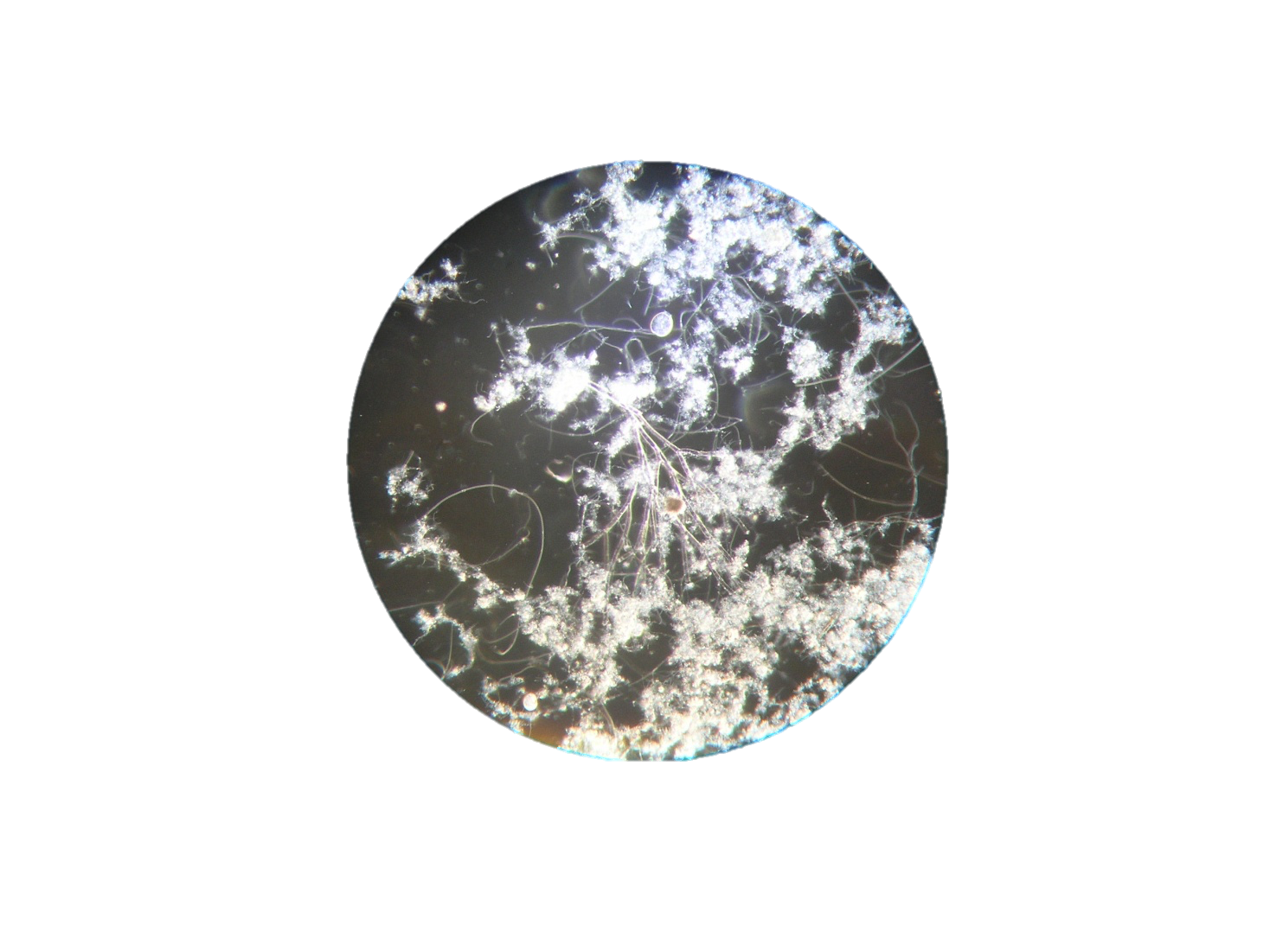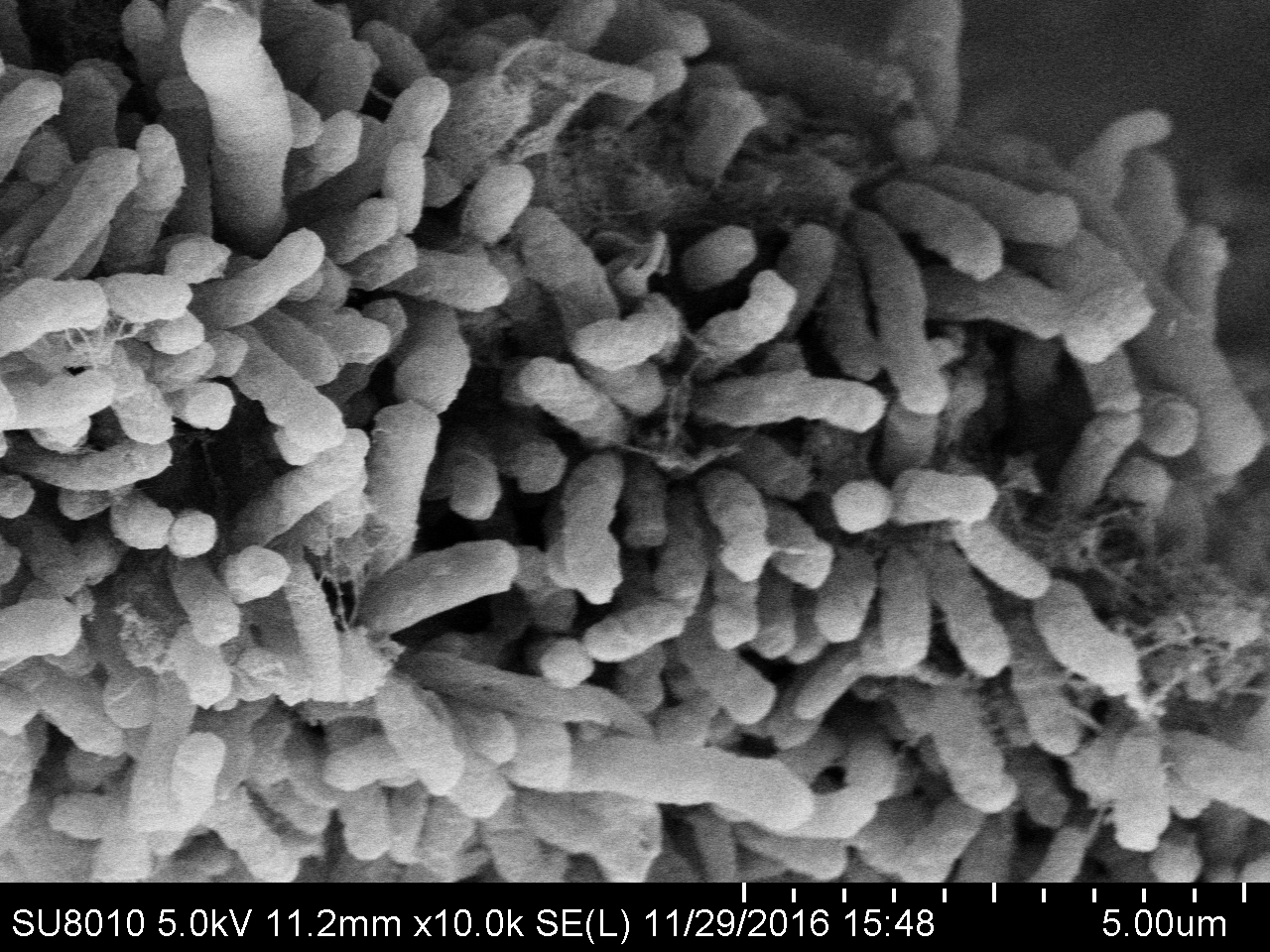 |  |
| --- | --- |
| (a)（×200） | (b)（×10.0k） |

**Fig. S1.** The microscopic appearance of sludge samples collected from the MAS process in Phase M1(a) and Phase M2(b).


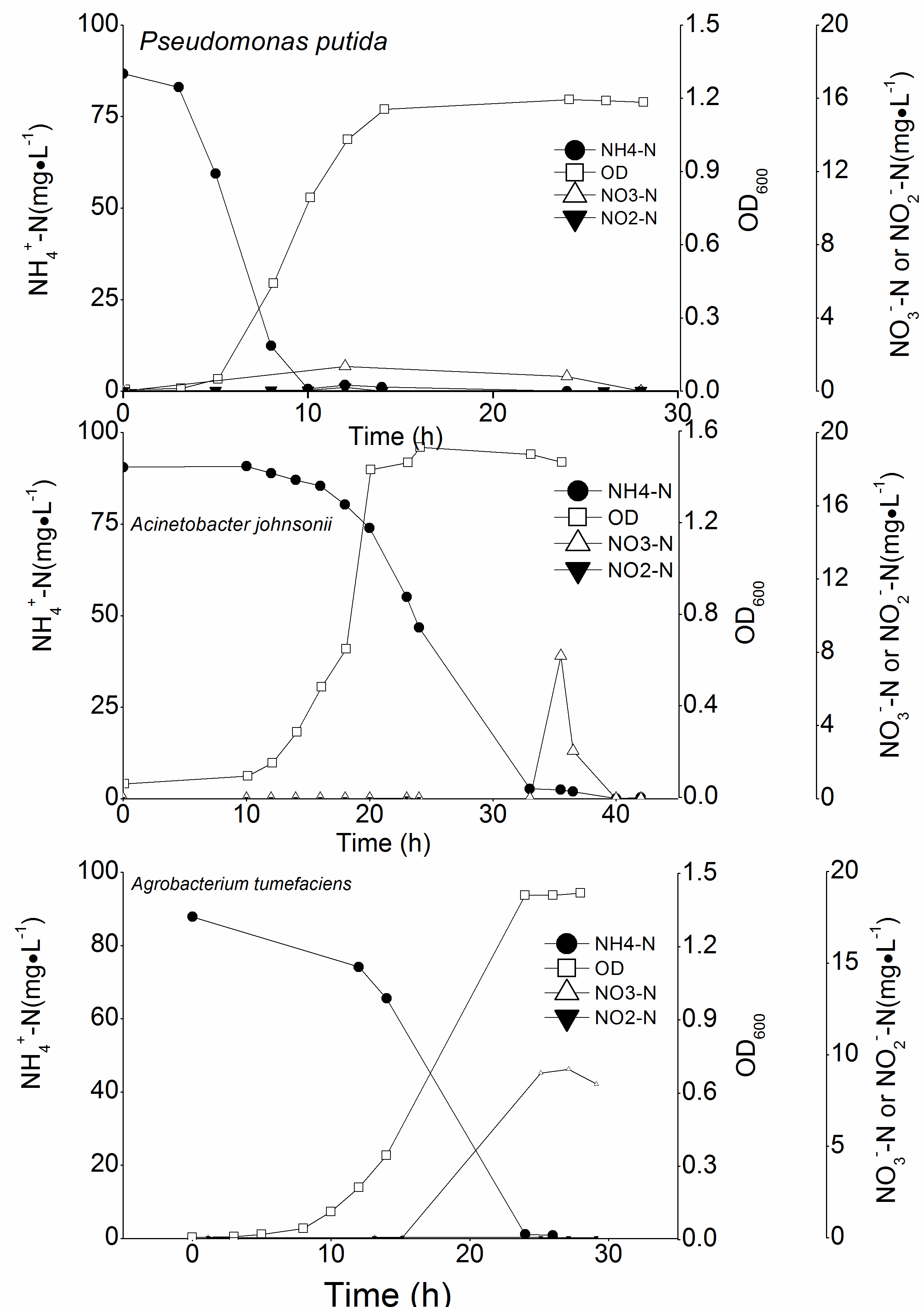


**Fig. S2.** Temporal variation of nitrogen compounds concentration and cell growth of OTU-identified isolates. OD: optical density.


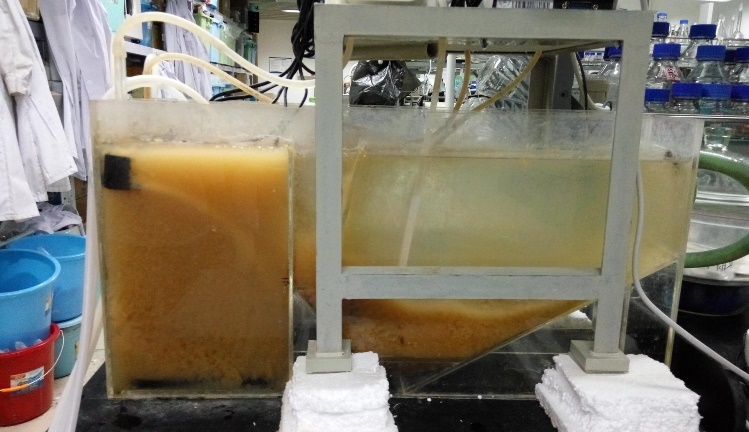


**aeration tank**

**clarification tank**

**Fig. S3.** The microaerobic activated sludge system used in this study.

**References**

Chen, Q., Ma, T., and Wang, T. (2014) Separation and Purification of Hydroxylamine Oxidase from *Agrobacterium tumefaciens LAD9.* *Biotechnology Bulletin* 69-73.

Li, M., Wang, Y., Zhao, S., Wang, Z., and Zhao, L. (2013) Characteristics of NH_4_^+^-N removal by *Acinetobacter johnsonii* at low temperature. *J Agro-Environ Sci* **32:** 2055-2060.

Padhi, S.K., Tripathy, S., Mohanty, S., and Maiti, N.K. (2017) Aerobic and heterotrophic nitrogen removal by *Enterobacter cloacae CF-S27* with efficient utilization of hydroxylamine. *Bioresour Technol* **232**: 285-296.

Chen, Q., and Ni, J. (2011) Heterotrophic nitrification-aerobic denitrification by novel isolated bacteria. *J Ind Microbiol Biotechnol* **38**: 1305-1310.

Preena, P.G., Manju, N.J., Deepesh, V., Thomas, A., and Singh, I.S.B. (2017) Genetic diversity of nitrate reducing bacteria in marine and brackish water nitrifying bacterial consortia generated for activating nitrifying bioreactors in recirculating aquaculture systems. *Aquac Res* **48**: 5729-5740.

Hun, L.B., Lee, M.G., Kim, J.K., 박경주, 조경숙, 김정보, and 홍영기 (2005) Isolation,identification and characterization of an immobilized bacterium producing N_2_ from NH_4_^+^ under an aerobic condition. *Environ Eng Res* **10:** 213-226.

Yang, L., Ren, Y., Zhao, S., Liang, X., and Wang J. (2016) Isolation and characterization of three heterotrophic nitrifying-aerobic denitrifying bacteria from a sequencing batch reactor. *Ann Microbiol* **66**:737-747.

1. [↑](#footnote-ref-1)
